# PRRT for well-differentiated gastroenteropancreatic neuroendocrine tumors (GEP-NETs)

**DOI:** 10.1530/ERC-25-0495

**Published:** 2026-03-23

**Authors:** Virginia Corbett, Garima Gupta, Aman Chauhan

**Affiliations:** ^1^ Division of Hematology/Oncology, Tisch Cancer Institute, Icahn School of Medicine at Mount Sinai, New York, New York, USA; ^2^ Division of Hematology/Oncology, University of Alabama at Birmingham, Birmingham, Alabama, USA; ^3^ Department of Hematology and Oncology, Helen Diller Family Comprehensive Cancer Center, UCSF, CA

**Keywords:** neuroendocrine tumors, peptide receptor radionuclide therapy, treatment sequencing, neuroendocrinology

## Abstract

Neuroendocrine tumors (NETs) are rare, heterogeneous neoplasms with varying prognoses and management approaches. Peptide receptor radionuclide therapy (PRRT), targeting tumors expressing somatostatin receptors, delivers cytotoxic radiation to tumor cells. While PRRT has demonstrated efficacy in advanced, well-differentiated NETs, the optimal sequencing with other therapies remains an area of active research. This review explores PRRT’s role in NET management, focusing on its mechanisms, clinical efficacy, safety profile, and integration into a multi-modal treatment strategy. We also examine evidence on the sequencing of PRRT with surgery, liver-directed therapy, chemotherapy, and targeted therapies to optimize treatment outcomes. We conducted a comprehensive review of recent clinical trials, cohort studies, and expert consensus guidelines to assess PRRT’s place in the treatment paradigm. Factors influencing treatment sequencing, such as tumor type, receptor expression, disease burden, and patient-specific characteristics, are also discussed. PRRT has proven effective for patients with advanced, somatostatin receptor-positive NETs, especially for inoperable tumors or those progressing after other therapies. While PRRT plays a vital role in management, its sequencing with other treatments remains complex, with evidence supporting its use both early and late in treatment based on individual patient factors. Optimizing its sequencing with other treatments requires further research but offers potential for improved outcomes. A personalized, multidisciplinary approach is essential for maximizing PRRT’s benefits in NET management. This review article summarizes current evidence and describes patient-specific circumstances, enabling treatment provider to make informed PRRT sequencing decisions.

## Introduction

Neuroendocrine neoplasms (NENs) are a highly diverse group of malignancies that can arise in several different organs throughout the body and can have significantly varying clinical behavior depending on the grade and secretory status of the tumor. The incidence and prevalence of NENs is rising with gastroenteropancreatic neuroendocrine neoplasms (GEP-NENs) comprising about 70% of cases, now being the second most prevalent gastrointestinal neoplasm after colon cancer (1, 2, 3). NENs include well-differentiated neuroendocrine tumors (WD-NETs), poorly differentiated neuroendocrine carcinomas (NECs), and mixed neuroendocrine–non-NENs. WD-NETs are subclassified into three grades based on the Ki-67 proliferation index and/or mitotic count: grade 1 (G1), grade 2 (G2), and grade 3 (G3) (4).

One of the unique characteristics of NENs is their expression of somatostatin receptors (SSTRs), which belong to a family of G-protein-coupled 7-transmembrane receptors and are expressed on majority of well-differentiated GEP-NETs (5). High-grade NETs and NECs can lose SSTR expression and are associated with a worse prognosis (6). The membranous expression of SSTRs has been leveraged for theranostic clinical applications with development of SSTR-targeting radioligands being used for functional imaging and treatment. Standard SSTR imaging techniques for NENs include radiolabeled DOTA-conjugated peptide (^68^Ga-DOTATATE, ^68^Ga-DOTATOC, or ^64^Cu-DOTATATE) for positron emission tomography/computed tomography (PET/CT) or PET/magnetic resonance imaging (MRI).

Nearly 50% of NET patients are diagnosed with synchronous distant metastasis at presentation (7). The treatment of advanced GEP-NETs is nuanced with limited studies establishing optimal sequencing. Treatment requires a multidisciplinary approach, and factors including tumor grade, burden and location, rate of progression, symptoms, and comorbidities play a key role in creating a treatment plan. Current systemic therapy options include somatostatin analogs (SSAs), capecitabine/temozolomide, radioligand therapy with [177Lu]Lu-DOTA-TATE (^177^Lu-DOTATATE) for somatostatin receptor 2 (SSTR2)-positive tumors, everolimus, and tyrosine kinase inhibitors (TKIs) (8). This review will provide an overview on the current role of peptide receptor radionuclide therapy (PRRT) in management of WD GEP-NETs and unique challenges in clinical practice, especially in view of recent advances, NETTER-2 and CABINET.

## Methods

This review includes significant studies establishing treatment standards for patients with WD GEP-NETs treated with PRRT. These papers were selected based upon their impact on the literature and relevance to the discussion. These papers were identified via expert opinion and through a literature search. Clinical trials discussed in this review were selected based on the opinion of the authors on their potential impact to the field.

## Outcomes with PRRT

PRRT with ^177^Lu-DOTATATE is a type of SSTR-targeting radionuclide therapy, which causes radiation-induced DNA damage and cell death through beta radiation-emitting properties of lutetium-177. The approval of PRRT in the United States in 2018 was largely driven by the publication of the NETTER-1 trial, which was a phase III trial comparing ^177^Lu-DOTATATE plus octreotide long-acting release (LAR) 30 mg once every 4 weeks to high-dose octreotide LAR (60 mg once every 4 weeks) in patients with advanced midgut SSTR-positive WD G1–G2 NETs (Ki-67 ≤ 20%) who had progression on standard octreotide LAR. 200 mCi of ^177^Lu-DOTATATE were administered activity every 8 weeks for a total of four cycles. The trial met its primary endpoint of prolonging progression-free survival (PFS), which was 8.4 months (95% CI: 5.8–9.1), with octreotide LAR alone, and the endpoint is not reached in the PRRT arm (*P* < 0.001; hazard ratio (HR) = 0.21; 95% CI: 0.13–0.33) (9). Overall survival (OS) analysis at five years revealed a median OS of 48 months in the PRRT arm compared to 36.3 months in the control group. The difference in OS was not statistically significant, most likely due to a high cross over rate to the PRRT arm (10).

Data from Europe in a single-arm phase II study of patients with GEP-NETs, bronchial NETs, and NETs of unknown primary treated with ^177^Lu-DOTATATE revealed a median PFS of 29 months and median OS of 63 months. Patients with pancreatic NETs had the longest median OS of 71 months (11). Interestingly, the objective response rate (ORR) in this study was 39% compared to 18% noted in NETTER-1 with only 1–2% patients achieving complete responses in both studies (9, 10, 11). Other prospective and retrospective studies have revealed similar response rates and survival outcomes (12, 13, 14, 15, 16, 17). Although requiring further validation, transcript assays known as the PRRT predictive quotient (PPQ) and NETest have been shown to predict responses to PRRT (18, 19, 20).

More recently, the NETTER-2 phase III trial has compared ^177^Lu-DOTATATE plus octreotide 30 mg LAR then octreotide 30 mg LAR every 4 weeks (treatment group) to high-dose octreotide 60 mg LAR every 4 weeks (control group) in patients with grade 2 (Ki-67 ≥ 10% and ≤20%) and grade 3 (Ki-67 > 20% and ≤55%) SSTR-positive advanced GEP-NETs. The study revealed a median PFS of 8.5 months (95% CI: 7.7–13.8) in the control group versus 22.8 months (19.4–not estimated) in the ^177^Lu-DOTATATE group (*P* < 0.0001; HR = 0.276; 95% CI: 0.182–0.418). The ORR was noted to be 43% in the study arm with consistent benefit across all subgroups, including both grades and primary site (21).

In addition to radiographic control, PRRT is also effective in controlling tumor-related symptoms. In health-related quality-of-life (QoL) analysis of NETTER-1, patients treated with ^177^Lu-DOTATATE had a significant improvement in time to QoL deterioration for global health, physical functioning, diarrhea, pain, body image, disease-related worries, and fatigue compared to patients treated with high-dose octreotide LAR (22). Similarly, Khan *et al.* reported an improvement in health-related QoL as well as performance status and symptoms in patients with GEP and bronchial NETs treated with PRRT (23). Other studies have also reported an improvement in symptoms in patients with functional pancreatic NETs (24) and refractory carcinoid symptoms in patients with small bowel NETs (25, 26). Of note, there exists a possibility of developing a flare in functional symptoms and rarely a hormonal crisis during or shortly after administration of PRRT (24, 25, 26, 27).

## Imaging considerations for PRRT

In order to be eligible for ^177^Lu-DOTATATE PRRT, SSTR imaging must be performed beforehand. SSTR scintigraphy used for inclusion criteria in NETTER-1 has now been replaced by SSTR-PET using DOTA-conjugated peptide (^68^Ga-DOTATATE, ^68^Ga-DOTATOC, or ^64^Cu-DOTATATE) and is now standard due to its substantially superior lesion detection rate (28). Positive uptake on SSTR-PET is defined as having a higher intensity of uptake or radiopharmaceutical concentration compared to normal organs, typically quantified using a PET measurement known as standardized uptake value (SUV). Although there are no specific cutoffs for uptake, a multidisciplinary discussion with the nuclear medicine team is necessary to determine whether there is sufficient positive uptake to have a meaningful benefit from PRRT in light of considering other potential treatment options as well. There are also retrospective studies to suggest that pretreatment SSTR--PET uptake can be both a predictive marker for response to PRRT (29) and a prognostic marker (30) for NET patients, although these findings have not been validated prospectively.

As previously mentioned, high-grade NENs can either lose SSTR expression or have baseline heterogeneous SSTR expression on imaging. Very rarely, WD low-grade NETs can also have absence of SSTR expression resulting in negative SSTR imaging (31). The increased proliferation rate and aggressive biology of high-grade NENs present an opportunity to use ^18^F-FDG PET/CT for accurate staging in cases where uptake on SSTR imaging is either negative or heterogeneous. Consequently, it is not surprising that high uptake on ^18^F-FDG PET/CT is associated with a lower ORR, PFS, and OS after treatment with PRRT (32) and a poor prognosis overall (33). While there is a lack of consensus on whether ^18^F-FDG PET should be obtained at baseline for patients with high-grade NENs, many NET oncologists will often obtain both SSTR and FDG PETs at diagnosis. Chan *et al.* proposed the NETPET grading system, which incorporates the degree of uptake on both SSTR and FDG PET/CT performed within a month of each other. In their study, the highest of five representing fluorodeoxyglucose (FDG) uptake > SSTR uptake demonstrated the lowest OS, setting the stage for its use as a potential prognostic imaging biomarker (34).

Typically, diagnostic imaging for response assessment is performed 1–3 months after completion of four doses of PRRT. Although not currently standard, there may be a role for individualized dosimetric assessments using post-^177^Lu-DOTATATE scintigraphy with SPECT/CT for treatment planning (35). This approach is being studied in several ongoing clinical trials (Table 1). Outside of dosimetric assessments, imaging between cycles is done every 3–4 months at some centers and if there is concern for clinical progression. It is important to recognize that a flare in functional symptoms and rarely a hormonal crisis can occur during or shortly after administration of PRRT (27, 36). A transient rise in tumor markers and liver function enzymes is also sometimes seen and needs to be considered when making decisions about obtaining mid-treatment imaging (11). There are no specific guidelines on whether to use anatomical or SSTR functional imaging for response assessment. In most cases, cross-sectional imaging with contrast-enhanced CT or MRI for liver predominant disease is sufficient. It is important to note the possibility of pseudo-progression on anatomical imaging during or shortly after completion of PRRT (37). This occurs due to a transient increase in tumor size, most likely due to radiation-induced inflammation and edema. Functional imaging can be performed in distinguishing pseudo-progression from true progression (38). Subsequent timing and choice of imaging depend on tumor biology, burden of disease, and response to PRRT.

**Table 1:** Select ongoing clinical trials using PRRT in neuroendocrine neoplasms.

NCI #	Trial name	Trial summary	Sponsor
NCT04917484	Dosimetry-based PRRT versus standard-dose PRRT with Lu-177-DOTATOC in NEN patients (DOBATOC)	RCT for patients undergoing PRRT comparing standard treatment to individualized doses of Lu-177-DOTATOC based on dosimetry	Aarhus University Hospital
NCT06395402	^177^Lu-DOTATATE modified delivery based on individualized dosimetry (LUMOD-ID)	RCT for patients undergoing PRRT comparing standard treatment to individualized doses of ^177^Lu-DOTATATE based on dosimetry	University of Iowa
NCT05247905	Comparing capecitabine and temozolomide in combination with ^177^Lu-DOTATATE in patients with advanced pancreatic neuroendocrine tumors	Randomized phase II trial comparing capecitabine and temozolomide to ^177^Lu-DOTATATE for the treatment of advanced pancreatic NETs	Alliance for Clinical Trials in Oncology
NCT04919226	^177^Lu-EDOTREOTIDE versus best standard of care in well-differentiated aggressive grade 2 and grade 3 gastroenteropancreatic neuroendocrine tumors (GEP-NETs) – COMPOSE (COMPOSE)	Phase III RCT of ^177^Lu-EDOTREOTIDE vs standard of care in grade 2/3 WD GEP-NETs and PNETs. This includes six cycles of PRRT treatment with ^177^Lu-EDOTREOTIDE with a 6 week interval between the first two cycles and then 8 week intervals for all other cycles	ITM Solucin GmbH, Germany
NCT03049189	Efficacy and safety of ^177^Lu-EDOTREOTIDE PRRT in GEP-NET patients (COMPETE)	Phase III RCT of ^177^Lu-EDOTREOTIDE vs everolimus in WD GEP-NETs and PNETs. ^177^Lu-EDOTREOTIDE is given every 90 days for a maximum of four cycles	ITM Solucin GmbH
NCT04609592	Study of PRRT in metastatic, World Health Organization (WHO) grade 1 or 2, SSTR-positive, GEP-NETs who are candidates for cytoreductive surgery	Phase I trial of neoadjuvant PRRT in patients who are candidates for cytoreductive surgery	Stanford University
NCT05870423	Improving peptide receptor radionuclide therapy with PARP inhibitors (PRRT-PARPis)	Phase I trial of olaparib with PRRT in patients with WD GEP-NETs who have progressed on primary PRRT	Erasmus Medical Center
NCT05724108	Testing the effectiveness of an anti-cancer drug, triapine, when used with targeted radiation-based treatment (^177^Lu-DOTATATE), compared to ^177^Lu-DOTATATE alone for metastatic neuroendocrine tumors	Phase II randomized control trial of triapine with PRRT vs PRRT alone in patients with well-differentiated neuroendocrine tumors	National Cancer Institute (NCI)
NCT05475210	^177^Lu-DOTA-EB-TATE in adult patients with advanced, well-differentiated neuroendocrine tumors	Phase I clinical trial of the safety and dosimetry profiles of ^177^Lu-DOTA-EB-TATE in patients with advanced GEP-NETs	Molecular Targeting Technologies, Inc., USA
NCT05178693	Lutathera and ASTX727 in neuroendocrine tumors (LANTana)	Phase I trial of ASTX727 in combination with PRRT	Imperial College London
NCT05053854	PARP inhibitor with ^177^Lu-DOTA-Octreotate PRRT in patients with neuroendocrine tumors (PARLuNET)	Phase I trial of talazoparib in combination with ^177^Lu-DOTA-Octreotate peptide receptor radionuclide therapy (PRRT)	Peter MacCallum Cancer Centre, Australia
NCT05477576	Study of RYZ101 compared with SOC in patients with inoperable SSTR + well-differentiated GEP-NETs that have progressed following ^177^Lu-SSA therapy (ACTION-1)	Study of RYZ101 compared with SOC in patients with inoperable SSTR-positive well-differentiated GEP-NETs that have progressed following ^177^Lu-SSA therapy (ACTION-1)	RayzeBio, Inc., USA
NCT05773274	Comparing retreatment of ^177^Lu-DOTATATE PRRT versus everolimus in patients with metastatic unresectable midgut neuroendocrine tumors (NET RETREAT trial)	Phase II trial of re-treatment with ^177^Lu-DOTATATE with standard-of-care treatment	National Cancer Institute (NCI)
NCT05153772	Targeted alpha-emitter therapy of PRRT-naïve and previous PRRT neuroendocrine tumor patients (ALPHAMEDIX02)	Phase II trial to evaluate the safety and effectiveness of 212Pb-DOTAMTATE in NETs	Radiomedix, Inc., USA
NCT06784752	Study to evaluate the efficacy and safety of [177Lu]Lu-DOTA-TATE in patients with grade 1 and grade 2 advanced GEP-NETs (NETTER-3)	Phase III randomized control trial of PRRT vs standard-of-care treatment in patients with grade 1 and grade 2 NETs	Novartis Pharmaceuticals

## Sequencing PRRT in advanced GEP-NETs

**Figure 1:**
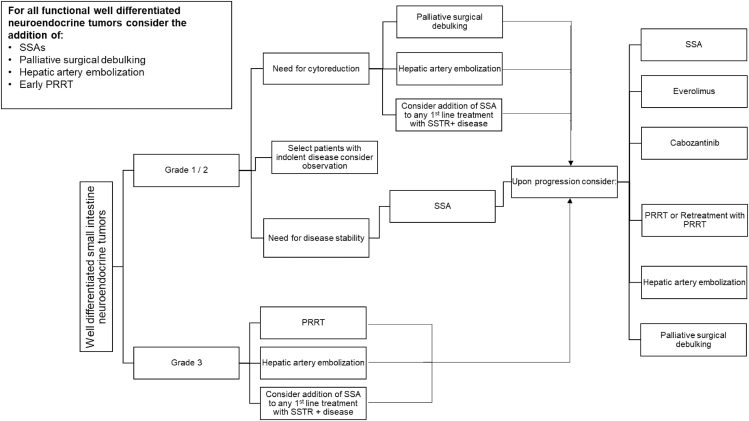
Treatment sequencing advanced well-differentiated small intestine neuroendocrine tumors.

**Figure 2:**
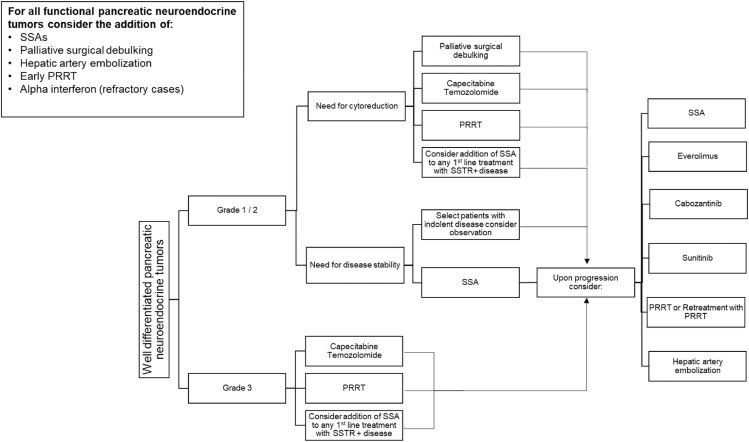
Treatment sequencing advanced well-differentiated pancreatic neuroendocrine tumors.

Of the treatment options for advanced GEP-NETs, timing and sequencing of PRRT should be evaluated in the context of several factors, including tumor biology, disease burden, primary as well as metastatic sites, need for tumor response, and comorbidities (39). Most studies on patients treated with ^177^Lu-DOTATATE has been after progression on first-line SSA until the NETTER-2 trial established a role for PRRT in the frontline setting for patients with advanced grade 2 or 3 disease. For most patients with advanced low-grade GEP-NETs, first-line treatment with SSA alone is effective in providing long-term symptom and disease control (40, 41). Surgical debulking and liver-directed therapy should be considered and discussed in a multidisciplinary setting (Figs 1 and 2).

For second-line treatment of low-grade GEP-NETs, there is not one optimal sequence and each patient’s treatment should be individualized and discussed in a multidisciplinary setting. Treatment options include everolimus, capecitabine/temozolomide, TKIs (sunitinib for pancreatic NETs and cabozantinib for midgut and pancreatic NETs), and ^177^Lu-DOTATATE. For patients with high-volume or symptomatic low-grade GEP-NETs, incorporating ^177^Lu-DOTATATE over everolimus and TKIs is reasonable. For patients where accelerated cytoreduction is required, sequencing capecitabine/temozolomide over other treatment options should be considered (42, 43, 44). For patients with low-grade midgut NETs, responses with capecitabine/temozolomide are not as impressive (42, 44, 45) and sequencing ^177^Lu-DOTATATE for symptomatic patients earlier is reasonable. Data suggest that sequencing ^177^Lu-based PRRT after everolimus (46) and cytotoxic chemotherapy (47) is safe and feasible in patients with GEP-NETs.

The NETTER-2 phase III trial showed an improvement in median PFS of 14 months with first-line ^177^Lu-DOTATATE plus octreotide 30 mg LAR every 4 weeks (treatment group) as compared to high-dose octreotide 60 mg LAR every 4 weeks (control group) in patients with grade 2 (Ki-67 ≥ 10% and ≤20%) and grade 3 (Ki-67 > 20% and ≤55%) SSTR-positive advanced GEP-NETs (21). High-grade NETs include a wide range of Ki-67 and can exhibit significantly varied biology, ranging from relatively indolent disease to rapidly progressive tumors. The impressive improvement in median PFS from the NETTER-2 trial should be carefully assessed along with the individual patient characteristics. First-line PRRT should be considered in this patient population; however, it may not be indicated in asymptomatic patients and patients with low symptom/disease burden and slower growth rate. For patients with rapidly progressive high-volume disease or patients in visceral crisis, cytotoxic chemotherapy should be considered over PRRT. There are also ongoing studies: NCT05247905 (^177^Lu-DOTATATE versus capecitabine/temozolomide in WD pancreatic NETs), NCT04919226 (^177^Lu-EDOTREOTIDE versus best standard of care in G2/G3 GEP-NETs), and NCT03049189 (^177^Lu-EDOTREOTIDE versus everolimus in G1/G2 GEP-NETs), which will help provide more evidence on sequencing of PRRT (Table 1).

## Unique challenges with PRRT

**Figure 3:**
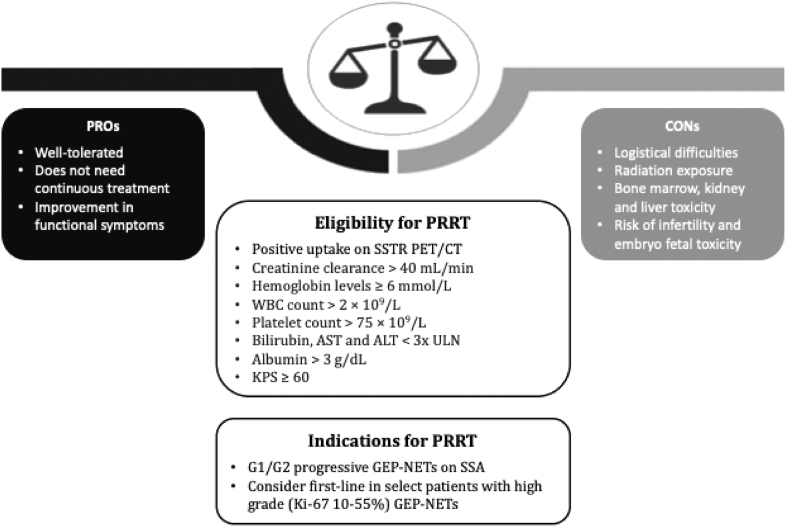
PRRT considerations.

When considering PRRT, it is critical to carefully individualize the treatment plan to each NET patient’s unique needs, including tumor biology, grade, prior treatment, and known tempo of the patient’s disease course. PRRT was approved based on the NETTER-1 study, which selected healthy patients with robust organ function. The exclusion criteria for NETTER-1 included patients with creatinine clearance of less than 50 mL/min and albumin >3. The study selected patients who will be less likely to experience known toxicities of radioligand therapy with particular emphasis on patients with normal kidney and liver function as indicated by creatinine clearance and hepatic function as indicated by albumin. In the real world, however, exceptions have to be made as patients who fall out of the specific parameters prescribed in the inclusion criteria of the NETTER-1 trial can benefit from PRRT. Understanding the impact of PRRT in unique situations, including decreased organ function, high disease burden, prior cytotoxic treatments, and prior radiation, can assist oncologists in tailoring treatment to each individual NET patient (Table 2, Fig. 3).

**Table 2:** Summary of challenges with PRRT.

Specific situation	Summary
CKD	PRRT is safe in most patients with CrCl > 30. Consider dose reduction and additional hydration. Hydronephrosis must be corrected prior to PRRT
Cytopenia or pre-existing CHIP	Hematologic toxicity is a potential risk with PRRT. Consider dose reduction and alternatives in high-risk patients. This is an ongoing area of active research
Peritoneal metastatic disease	PRRT is well tolerated even with peritoneal disease. For those at highest risks, consider prophylactic steroids post-treatment
High-volume liver metastasis	PRRT is well tolerated in many patients with a high burden of metastatic liver disease. Liver function is a more important parameter of the risk of hepatic toxicity rather than volume or disease burden in the liver
Sequencing with prior Y90	Patients with high volume of prior Y90 radioembolization treatment may be at a higher risk of hepatotoxicity after PRRT
Re-treatment with PRRT	Salvage PRRT with two additional cycles can be done safely and effectively in patients who have received an initial four cycles of PRRT

### PRRT in patients with chronic kidney disease

Concern for renal toxicity of PRRT is based on the risk of update of radioligand peptides in the proximal tubule of the kidney (48, 49). In the setting of a reduced GFR, there is an increased risk that absorbed radioligand peptide uptake may persist in the kidney, resulting in tissue damage and leading to radiation-induced renal thrombotic microangiopathy and renal failure (50). In historical studies of ^90^yttrium-based radioligand therapy, significant renal toxicity occurred in as high as 10% of patients (50, 51). The addition of routine amino acid infusion standard-of-care therapy with PRRT has significantly reduced this risk as the amino acids compete for uptake of peptides in the proximal tubule preventing radiation-induced nephrotoxicity (51, 52). In recent studies using the FDA-approved ^177^Lu-DOTATATE administered with standard-of-care renal protective amino acid infusions, the risk of acute kidney toxicity in patients with creatinine clearance >50 mL/min is low (52, 53).

An important question remains for patients with creatinine clearance <50 mL/min. There is concern that with decreasing renal function, increased radioligand peptide (54) is absorbed in the proximal tubule increasing the risk of radiation-induced nephrotoxicity. In subgroup analysis of NETTER-1, it was noted that in the ^177^Lu-DOTATATE arm, there were 11 patients with mild baseline renal dysfunction with CrCl 50–60 and 13 patients with moderate dysfunction with CrCl < 50 mL/min. Those patients with mild or moderate renal dysfunction had no increased risk of renal toxicity and decompensation of renal function over time (55). Another prospective study of ^177^Lu-DOTATATE in 51 patients found that the median creatinine for the entire group increased from baseline 1 year after treatment. Seventeen patients had a more severe reduction in creatinine clearance of 20% or greater. Patients with other risk factors for CKD, including hypertension and diabetes, were at a highest risk of renal decompensation with PRRT treatment. The authors described one patient in the group with a more severe decline in renal function after PRRT associated with hydronephrosis (12).

Patients with NENs, particularly midgut NENs are at an increased risk of hydronephrosis related to metastatic tumor deposits or related to mesenteric fibrosis (56). The risk of renal toxicity in patients with hydronephrosis is high given the pooling of radioligand particles and associated radiation exposure. Patients with hydronephrosis require close collaboration with urology and correction of hydronephrosis prior to PRRT to prevent radiation exposure and permanent renal toxicity (57, 58).

In addition, retrospective studies have also evaluated the risk of renal decompensation in patients receiving PRRT. One single-center retrospective analysis identified 33 patients with an estimated glomerular filtration rate (eGFR) of less than 60 mL/min/1.73 m^2^, with 26 with more severe kidney disease with a GFR of 45–60 mL/min/1.73 m^2^ and 7 with CKD with a GFR of 30–45 mL/min/1.73 m^2^. In the group with a GFR of 30–45 mL/min/1.73 m^2^, only one patient (3%) developed permanent grade 4 nephrotoxicity. When all subgroups were considered, there was an association between PRRT and a slight decrease in GFR in 15 patients (GFR worsening by less than 10 after PRRT) and nine patients moving into higher CKD groups demonstrating slight worsening of renal function (59, 60). Another retrospective study included 39 patients with known CKD, including three patients with grade 4 CKD (eGFR 20–30 mL/min). Patients with CKD had worse hematologic toxicity with PRRT treatment (60, 61).

Nephrotoxicity is rare in most patients treated with PRRT. In patients with CKD, however, PRRT may increase the risk of worsening CKD and hematologic toxicity. The prescribing information for PRRT states that ‘no dose adjustment is recommended for patients with baseline mild to moderate (creatinine clearance 30–89 mL/min by Cockcroft–Gault formula) renal impairment’. However, it does highlight the risk of greater possible renal toxicity due to a higher absorbed dose (61, 62). Most guidelines suggest that a CrCl < 50 mL/min is not an absolute contraindication for PRRT. In clinical practice, additional fluids can be considered before and after PRRT to decrease the risk of kidney injury in patients with borderline kidney function. Use of PRRT in patients with CrCl ≤ 30 is high risk and, in most cases, not recommended (57, 58). Of note, PRRT can be used safely in the setting of hemodialysis. Most often, PRRT in this setting is given at a reduced dose and in close collaboration with nephrology and institutional radiation safety officers to ensure appropriately timed hemodialysis after radioligand infusion (62, 63, 64).

### PRRT in patients with peritoneal disease

Small bowel obstruction related to primary disease, peritoneal implantation, mesenteric masses, and/or fibrosis is a known complication of midgut NETs (56, 65). Radiation can cause enteritis, which can lead to severe side effects for patients, including risk of diarrhea, abdominal pain, and digestive issues including weight loss (66, 67). In addition, many patients with small bowel NETs undergo surgical resection of their small bowel, leading to an increased risk of obstruction due to postoperative adhesions. PRRT itself has potential to cause pseudoprogression as radiation-induced inflammation can lead to tumor edema (37). The risk of intestinal obstruction with PRRT was retrospectively reviewed in 189 patients. In this study, 81 patients had peritoneal or mesenteric disease, of which 22 were noted to be at a very high risk of complications, including possible obstruction, given extensive mesenteric or peritoneal disease and tumor-associated desmoplastic changes and fibrosis. In the population as a whole, five patients (3%) experienced intestinal obstruction within 3 months of PRRT. Several of them had a significant improvement in symptoms with steroid treatment, commonly with dexamethasone. Two patients died after PRRT treatment related to obstruction, and another required parental nutrition (67, 68). Another retrospective review of 80 patients described an additional four patients with PRRT-related intestinal obstruction (68, 69).

In patients with extensive peritoneal disease, intestinal obstruction is a risk with PRRT treatment. In most patients, this resolves often with steroids, but in a small group of patients, this can be life-threatening. Given this finding, for patients at a high risk of intestinal obstruction, many centers will prescribe prophylactic steroids to be given after PRRT treatment. It is important to note that steroids should not be given prior to PRRT treatment as this may reduce the expression of SSTR2, making PRRT less effective (69, 70). One to 2 weeks of steroids administered after PRRT may reduce the risk of obstruction in the period where inflammatory changes from PRRT are at their peak (57, 58).

### Prior chemotherapy and preleukemic myeloid mutations

The development of secondary myeloid neoplasms is a feared complication of PRRT. In the final analysis of NETTER-1, 2% of patients developed secondary therapy-related myeloid neoplasms (t-MNs), one of which led to a patient death (70, 71). In several large clinical retrospective cohorts, the incidence of t-MNs after PRRT ranged from 1.8 to 4.8% (11, 71, 72). Other groups have reported higher numbers, particularly in patients with prior chemotherapy with alkylating agents and Y90-based radioligand therapy (73, 74). Risk factors for the development of t-MNs post-PRRT include heavy burden of disease in the bone marrow, prolonged thrombocytopenia, and longer duration of PRRT treatment (52, 53). Recent studies have suggested that the risk of t-MNs post-PRRT may be mediated by clonal hematopoiesis of indeterminate potential (CHIP) (75, 76, 77). CHIP is the presence of preleukemic myeloid mutations in patients without known myeloid disease and is normally associated with aging (78). CHIP mutations induce a pro-inflammatory state and are associated not only with a risk of development of myeloid neoplasms but also with a high risk of cardiovascular disease, kidney disease, diabetes, and other inflammatory diseases of aging (79, 80, 81, 82, 83).

Next-generation sequencing (NGS) testing of circulating tumor cells (CTCs) used in several solid tumor types has recently become more popular in WD NETs (84). NGS of CTS from peripheral blood for solid tumors is capable of picking up CHIP mutations, including those associated with an increased risk of t-MNs (79, 80, 81, 82, 83, 85). The decision about when and if to pursue PRRT in a patient with a WD NET is complex. However, the risk of t-MNs is a real concern for patients, and although the risk is small, the consequences are severe. In patients with known risk factors, such as prior alkylating chemotherapy and/or large volume of bony metastasis, some experts have suggested starting at a lower dose of radioligand therapy (58, 59). For patients with known CHIP mutations found on routine NGS testing, there are currently no clear recommendations on how to proceed with PRRT. A risk and benefit discussion should be held with patients for any high-risk patient, optimally in coordination with hematology prior to consideration for PRRT.

### Thrombocytopenia and PRRT

Thrombocytopenia is a notable adverse effect of PRRT, and management of thrombocytopenia requires additional attention. In both the NETTER-1 and NETTER-2 trials, grade 3 or 4 thrombocytopenia occurred in only 2% of enrolled patients (21, 86). The prescribing information for PRRT with ^177^Lu-DOTATATE suggests that if significant thrombocytopenia occurs during the course of treatment, prescribers should withhold the next radionuclide dose until complete or partial resolution and reduce the dose by half for the following cycle. For severe or refractory thrombocytopenia of grade 2 or higher or if there is a delay in treatment greater than 16 weeks, permanently discontinuing PRRT is recommended (61). Although the vast majority of cases of PRRT-associated thrombocytopenia are reversible, in rare cases, refractory thrombocytopenia can occur. In a large cohort of 203 patients treated with PRRT, reversible grade 3 or 4 thrombocytopenia occurred in 1.7% of patients. The mean time for normalization of cytopenias was 12 months post-PRRT. Interestingly, in this study, splenectomy was inversely correlated with the development of thrombocytopenia or leukopenia (87). Another study evaluated three patients with severe and persistent cytopenias after or during PRRT treatment. All three patients required dose reduction or cessation of PRRT due to thrombocytopenia. In this study, each patient was found to have extensive bone metastasis prior to PRRT treatment and bone marrow biopsies were done for each patient to rule out secondary hematologic malignancy. For each patient, bone marrow biopsy revealed extensive metastatic disease (88). Patients with bone metastasis prior to PRRT should be monitored closely for the development of thrombocytopenia post-treatment. As described previously, CHIP mutations are common in NET patients and may predict the risk of the development of t-MNs after treatment. In a study of 37 patients who underwent evaluation for CHIP prior to PRRT, the presence of CHIP mutations was associated with an increased risk of thrombocytopenia prior to treatment and more prolonged thrombocytopenia post-treatment. In addition to evaluation for bony metastasis, the presence of CHIP may be considered a risk factor for prolonged thrombocytopenia post-treatment (77).

### PRRT in patients with high volume of metastatic liver disease

Liver metastases are common in patients with WD NETs (87, 89). Radiation-induced liver toxicity is a potentially life-threatening consequence of radioligand therapy. Due to concern for hepatic toxicity, the major phase III clinical trials using ^177^Lu-DOTATATE, including NETTER-1 and NETTER-2, excluded patients with liver dysfunction, defined by total bilirubin > 3× ULN and serum albumin <3.0 g/dL. Historically, there has been concern that an increased tumor burden in the liver would increase the risk of off-target radiation toxicity with radioligand therapy. However, in several large retrospective studies, PRRT has proven safe and effective, even in patients with a large volume of metastatic liver disease. In one large study of 371 patients receiving PRRT, 15 patients were included with large-volume liver disease, defined as >75% of tumor liver involvement. Of these, only one patient experienced mild hepatotoxicity, and the remainder tolerated treatment well (88, 90). In other large retrospective studies with PRRT, the incidence of hepatotoxicity was rare (9, 11). In practice, the risks and benefits of PRRT should be assessed with total liver disease burden and with liver health and function as even patients with a high degree of metastatic liver involvement can continue to have robust liver function and can tolerate treatment well.

### Re-treatment or salvage PRRT

Given the limited treatment options for patients with WD NENs, re-treatment with PRRT remains an excellent option in select patients. Patients who have progressed on standard treatment including the initial four cycles of PRRT can be considered for an additional two cycles of PRRT. Retrospective studies have evaluated salvage PRRT, including a large retrospective study of 168 patients treated with salvage PRRT and a smaller population with an additional re-treatment beyond six cycles. In this group, the median PFS after re-treatment with PRRT was 14.6 months. No grade 3 or higher renal toxicity was observed, and 2.2% patients developed t-MNs after treatment (89, 91). Other smaller retrospective studies have also explored this topic (90, 91, 92, 93). A systematic review explored these studies and determined that salvage PRRT had an ORR of 17% and a median PFS of 14.1 months. In pooled analysis, the rate of hematologic toxicity was 10% and the rate of nephrotoxicity was 0.7% (92, 94). A prospective phase II clinical trial evaluating the role of re-treatment PRRT is currently ongoing (Table 1). In patients who have exhausted all FDA-approved treatment options, re-treatment with PRRT remains an excellent option with strong retrospective data demonstrating safety and efficacy. If re-treatment PRRT is pursued, enrollment on the clinical trial is preferred if available. Currently, two randomized prospective clinical trials are investigating PRRT re-treatment strategies. In the ACTION-1 study investigating alpha-PRRT after standard-of-care ^177^Lu-DOTATATE treatment, patients who have had stable disease for at least 6 months post-PRRT are eligible for re-treatment with an alpha-PRRT investigation agent. This study has now completed enrollment, and results are expected in the next 1–2 years (NCT05477576). NET-RETREAT is a randomized phase II clinical trial evaluating re-treatment of ^177^Lu-DOTATATE vs standard-of-care everolimus, sunitinib, or cabozantinib in patients who have previously been treated with ^177^Lu-DOTATATE and benefited for at least 12 months post-initial PRRT (95). This is a federally funded study currently enrolling in over 25 sites in the US and Canada. Both these studies have potential to change practice and may formalize the role of PRRT re-challenge at progression.

### Neoadjuvant PRRT

The role of neoadjuvant PRRT in well-differentiated GEP-NETs has been explored in several retrospective studies, whereas prospective data remain limited. A scoping review describing the existing literature on the role of neoadjuvant PRRT in patients with initially unresectable GEP-NENs identified eight case reports, eight retrospective cohort studies, and one ongoing phase II clinical trial (93, 96). These included both ^177^Lu and ^90^Y isotopes alone or in combination with radiosensitizing chemotherapy. In the largest retrospective cohort of 57 patients with unresectable GEP-NETs with or without liver metastasis who received 2–5 cycles of ^177^Lu-DOTATATE, the primary tumor became resectable in 15 of 57 patients (94, 97).

Neoadjuvant PRRT in pancreatic NETs has been an area of active investigation. The largest retrospective cohort examining neoadjuvant PRRT in pancreatic NETs included 29 patients with non-functioning tumors treated in the Erasmus University Medical Center. All patients included had imaging showing tumors that were borderline resectable or had limited, potentially resectable metastatic disease. PRRT was given to all patients. Of the initial 29 patients, nine patients went on to undergo surgical resection of their tumor. In this study, the median PFS was improved at 69 months for the small number of patients who underwent surgery vs 49 months for all other patients (95, 98). Another retrospective study of 110 patients treated with PRRT identified nine cases where PRRT was given in the neoadjuvant setting with the intent to make borderline resectable patients eligible for surgical resection. Of these patients, four became eligible for surgical resection after neoadjuvant PRRT (96, 99). Another smaller study included 23 patients with resectable or potentially resectable pancreatic NETs with high-risk features (radiologic tumor size >4 cm and presence of nearby organ or vascular involvement and/or resectable or potentially resectable liver metastases) who received neoadjuvant ^177^Lu-DOTATATE, and outcomes were compared to matched patients who underwent frontline surgery. In this study, there was no difference in PFS or disease-specific survival between the two groups (97, 100). These small retrospective studies are difficult to interpret due to variation in ^177^Lu-DOTATATE dosing and the lack of standardization of patient selection for neoadjuvant PRRT.

The utility of neoadjuvant PRRT in pancreatic NENs was explored prospectively in a phase II clinical trial, the ‘NEOLUPANET study’, a multi-site, open label, single-arm study, which included 31 patients at 8 Italian institutions. This study included patients with well-differentiated, non-functioning pancreatic NETs with at least one high-risk feature, including included tumor size >4 cm, enlarged nodes on imaging, single liver metastasis, Ki-67 > 10%, vascular invasion, vein thrombosis, and nearby organ involvement. The primary endpoint was safety of surgery after PRRT – including assessment of risk of complications and death within 90 days of resection. Twenty-six of 31 patients were able to complete all four cycles of PRRT, and 29 underwent surgery with primary tumor resection. One patient was found to have unresectable disease at the time of surgery due to extensive vascular infiltration. The ORR was 58% with no complete responses and no patients with progressive disease. Twenty-four patients were able to achieve a R0 resection and four patients R1. The surgical outcomes and morbidity and mortality were comparable to contemporary outcomes. The main takeaways from this trial include prospective data demonstrating the safety of resection after PRRT and the efficacy of PRRT in generating partial responses in the neoadjuvant setting (98, 101).

An additional prospective study designed to explore the safety and efficacy of neoadjuvant PRRT is ongoing. This is a phase I study (NCT04609592) looking at the utility of PRRT in all patients with gastroenteropancreatic NETs who are eligible for cytoreductive surgery. Patients with metastatic disease who are candidates for debulking surgery can be enrolled. The study plan includes two cycles of PRRT followed by cytoreductive surgery, followed by up to two additional cycles of PRRT for residual disease as determined by post-procedure ^68^Ga-DOTATATE PET/CT. The primary endpoints include safety and feasibility of neoadjuvant PRRT prior to surgical debulking (Table 1).

PRRT is an important tool for symptom and disease control in NENs. In select patients, PRRT can be considered as a potential tool for cytoreduction. However, further studies are needed to confirm the safety and efficacy of this in practice and details regarding its impact on recurrence rates, long-term toxicity and survival, and primary site and to compare it to systemic therapy options that may be used in the neoadjuvant setting. In addition, the issue of radiation-induced fibrosis potentially complicating surgery is a concern for patients who undergo neoadjuvant PRRT, and it is recommended that consideration of neoadjuvant PRRT for pancreatic NETs is best done in experienced centers. Although further studies are needed to better explore the role of neoadjuvant PRRT prior to surgery, there is recent retrospective evidence that suggests debulking surgery prior to PRRT may enhance the efficacy of PRRT by reducing the total tumor burden (99, 102).

### Y90 and PRRT sequencing

Y90 radioembolization is an effective treatment modality in select patients with WD NETs with liver metastatic disease and, as per NCCN guidelines, has an important role in patients with lobar, segmental disease and in patients with a colonized biliary tract due to prior procedures (100, 103). Liver-directed therapy with Y90 can result in radiation-induced liver damage, which can be noted years after the initial treatment and is thought to be related to radiation-induced injury of normal liver parenchyma (101, 102, 104, 105). This risk is greatest in patients treated with bilobar Y90 radioembolization (103, 104, 106, 107). Although Y90 is widely used in clinical practice, several retrospective studies have suggested an increased risk of radiation-induced liver failure in patients who receive PRRT after Y90 therapy. In one retrospective analysis including 17 patients receiving PRRT, 35.3% of patients received locoregional therapy with Y90 prior to PRRT. In the study, 41% of patients developed ascites after PRRT and 3 (18%) died of liver failure (105, 108). With a more recent understanding of the long-term risks of Y90 radioembolization, few patients these days are treated with bilobar therapy and techniques for dosimetry are more sophisticated allowing Y90 radioembolization to serve as targeted therapy for some patients with metastatic NETs. As clinicians evaluate patients for potential PRRT treatment, it is critical to consider history of Y90 radioembolization treatment and carefully consider the extent of prior radioembolization, the patient’s liver function, available imaging of the liver, and risk of liver decompensation as part of the risk–benefit analysis with PRRT. In patients with advanced SSTR-positive WD NETs who may be candidates for future PRRT, Y90 radioembolization should be done in the context of multidisciplinary discussion with IR, medical oncology, and nuclear medicine.

### PRRT in the elderly

While the incidence of NETs in patients 65 years or older rose by eightfold between 1973 and 2012 (1, 2, 3, 109), there remain limited data on the characteristics, risk factors, treatment patterns, and prognosis in this patient population (110), which showed that patients ≥70 years old with advanced NETs receive fewer treatments and have worse survival outcomes (5.2 vs 9.6 years) compared to patients <70 years old, with 71% dying from disease progression (110). Another single-institution study including 145 NET patients ≥70 years old reported a similar median OS of 5.1 years (111).

There are even more scant data on PRRT in elderly patients with advanced NETs. Elderly patients have a higher frequency of comorbidities and lower bone marrow reserve compared to younger patients. This introduces unique challenges to treatment options and sequencing in this patient population. The average age of patients enrolled in NETTER-1 and NETTER-2 was 63 and 61, respectively. Retrospective studies evaluating PRRT in elderly patients have not identified new safety signals and have demonstrated a similar toxicity profile and non-inferior survival outcomes compared to younger patients (111). In clinical practice, PRRT can be offered safely to elderly patients with NETs in the absence of any known contraindications. Prospective trials are required to better assess the efficacy and adverse effects associated with PRRT in elderly patients.

## Future directions

Although PRRT was first established as a standard treatment in the later-line setting, many recent studies have demonstrated the utility of PRRT in the frontline setting. As mentioned above, the NETTER-2 trial established an impressive improvement in median PFS in patients with grade 2 or grade 3 NETs with PRRT treatment in the frontline setting (21). In addition, the ongoing COMPOSE trial of ^177^Lu-EDOTREOTIDE in grade 2 and grade 3 NETs is notable for use of PRRT in the frontline setting, decreased time between treatment in the first two cycles, and additional treatment cycles for a total of six cycles vs standard treatment with four cycles (112). The NETTER-3 trial (NCT06784752) is also now planned, which is a phase III trial evaluating the use of PRRT versus standard of care in patients with grade 1 and grade 2 GEP-NETs. With the results of these subsequent studies, the sequencing of PRRT may change, with increased utility in the frontline setting and a possible rule for additional treatment cycles in select patients.

Combination therapies with PRRT are an area of active investigation to improve the efficacy of PRRT. Prior studies have evaluated combination therapies with PRRT in WD NETs (106, 113). Key studies include the combination of PRRT with 5-FU chemotherapy (107, 114), capecitabine (108, 115), capecitabine with temozolomide (109, 116), and everolimus (110, 117). Many of these combination therapies were associated with an increased risk of toxicity, particularly with increased hematologic toxicity. Preclinical data have demonstrated synergy between PARP inhibitors and PRRT by potentiating the effects of PRRT in developing and preventing repair of radiation-induced DNA double-strand breaks (DSBs) (111, 112, 118, 119).

Ongoing clinical trials are exploring the role of combination therapy of PARP inhibitors and PRRT. This includes early-phase trials of olaparib with PRRT (NCT05870423) and talazoparib (NCT05053854). Another promising study is examining the combination of the ribonucleotide reductase (RNR) triapine with PRRT. The RNR pathway is critically involved in pathways mediating repair of DSB in DNA, and by blocking DNA repair, the investigators hope to potentiate PRRT preventing repair of radiation-induced DNA damage and inducing greater cell death and treatment response (NCT05724108) (113, 120). Data from a multi-center phase I trial of triapine and ^177^Lu-DOTATATE have recently been presented at ESMO 2025 and look safe and promising. The combination is currently being investigated in a multi-center randomized phase II clinical trial (ETCTN 10558) and has completed enrollment (121).

Another such novel combination with DNA PK inhibitor called peposertib is currently being investigated in a multi-center clinical trial called ETCTN 10450. The expansion phase of the phase I study has completed enrollment, and the efficacy data are expected to be presented in the first quarter of 2026. Previously recommended phase II dose of peposertib in combination with ^177^Lu-DOTATATE was presented at NANETS 2023 (95).

Combinations with PRRT and immunotherapy have been explored in preclinical models, with promising early data suggesting synergy between potentially immunogenic radioligand therapy and checkpoint blockade with pembrolizumab (114, 122). This combination is promising in the treatment of the high-grade neuroendocrine cancer Merkel cell carcinoma (115, 116, 123, 124). PRRT with immunotherapy in G2/G3 WD NETs was shown to be well tolerated with 4 of 13 evaluable patients with responses (117, 125). The potential synergy of radiation-induced cancer cell death with immunotherapy is an area of active investigation (118, 126), and it remains to be seen if this synergy will be observed for patients with WD NETs.

Alpha-emitter PRRT (sometimes called targeted alpha-particle therapy (TRT)) is also an important area of active investigation revolutionizing cancer-directed theranostics. The current and historical standard of care included yttrium-90- and now lutetium-177-based radioligand therapy, both using β-emitting radioisotopes. Alpha-emitting radioisotopes represent the next generation of radioligand-based therapy. Radioisotope efficacy is mediated by path length and linear energy transfer. β-emitting radioisotopes have a reasonably long particle path length (≤12 mm) and low linear energy transfer (LET) (∼0.2 keV/μm), while alpha-emitting radioisotopes have a smaller path length (50–100 μm) and higher LET (80 keV/μm) (119, 127). Alpha-emitting anti-cancer radioisotopes are able to better target cancer cells due to higher LET and increased double-stranded DNA damage, with less collateral damage to surrounding healthy tissue due to a smaller path length (120, 128). Alpha-emitter-based radioligand therapy with ^212^Pb-DOTAMTATE and ^225^Ac-DOTATATE is already being examined in WD NETs in clinical trials (120, 128).

^212^Pb-DOTAMTATE was evaluated in preclinical studies using mice models, which revealed improved survival with low-dose ^212^Pb-DOTAMTATE with promising safety data (121, 129). ^212^Pb-DOTAMTATE was first explored in humans in a phase I study, including 20 patients with SSTR-positive WD NETs from all primary sites. Patients who had received prior PRRT were included. In this dose-finding study, ^212^Pb-DOTAMTATE was given at 8-week intervals for four cycles total. Treatment was well tolerated with an improvement in tumor burden, quality of life, pain levels, and few side effects (122, 130). ^212^Pb-DOTAMTATE was explored further in a phase II, open label, multi-center study. The primary end point of this study was ORR and incidence and severity of adverse events. This study included multiple cohorts, such as cohort 1 with 36 patients and cohort 2 with 30 patients, all with SSTR-positive advanced GEP-NETs. ^212^Pb-DOTAMTATE was administered at 67.6 μCi/kg for four cycles in total. In cohort 1, 17 of 36 (47.2%) patients had confirmed responses. Lymphocytopenia was a common adverse event with 69% of patients experiencing grade 3 or 4 lymphopenia. Surprisingly, there were several deaths among study participants with four fatal adverse events attributed to progressive disease, carcinoid syndrome, and sepsis (123, 131). Further studies are planned to better explore the safety and efficacy of ^212^Pb-DOTAMTATE radioligand therapy in WD NETs.

Preclinical studies of ^225^Ac-DOTATATE demonstrate effective targeting of SSTR-positive tumors. Renal toxicity was observed with higher doses of radiation (activities higher than 30 kBq induced kidney injury) (124, 132). ^225^Ac-DOTATATE has been explored in metastatic SSTR-positive WD NETs in a phase Ib trial dose de-escalation study that included 9 patients who had previously received standard PRRT with ^177^Lu-DOTATATE for four cycles. In this trial, patients received ^225^Ac-DOTATATE at a starting dose of 120 kBq/kg every 8 weeks for up to four treatments. No dose-limiting toxicities or treatment-related serious AEs were noted. One serious adverse event occurred, which was considered unrelated to the study drug (125, 133). Given these promising early data establishing safety of this agent, an ongoing phase III randomized control trial (ACTION-1) is exploring four cycles of ^225^Ac-DOTATATE compared to the investigator’s choice of standard-of-care treatment. The primary outcome of this trial will be PFS (NCT05477576). Although the results of this randomized control trial are still pending, there are additional data available about the ^225^Ac-DOTATATE product in advanced WD NETs from another study. A prospective, single-center study examined long-term outcomes of ^225^Ac-DOTATATE-based radioligand therapy. This study included 91 patients with advanced SSTR-positive tumors, with and without prior PRRT. In this study, capecitabine was given with each cycle of treatment as a radiosensitizer and the ORR was 44%. There were few grade 3 and 4 events; however, the study did describe malignant ascites contributing to death in ten patients after ^225^Ac-DOTATATE therapy (126, 134). Although the specific etiology of liver decompensation is not clear as both radiation-induced toxicity and progression of disease can contribute to ascites, these results do present a signal of risk of liver toxicity, which should be explored in greater detail in future trials. Of note, although this is a prospective trial, prior authors have questioned the rigor of this trial specifically due to concerns for shifting eligibility criteria, evaluation of response, and the inclusion of concurrent capecitabine chemotherapy (127, 135). ^225^Ac-DOTATATE is a promising agent in WD NETs, and further results are awaited, including the results of the ACTION-1 trial, to truly establish the response and safety profile of ^225^Ac-DOTATATE in patients with advanced NENs previously treated with PRRT.

## Conclusion

PRRT is a powerful treatment modality in WD NENs. The development of theranostics from its origins in Rotterdam in the 1980s (128, 136) has changed the treatment landscape for patients with WD NENs with significant improvements in quality of life, symptom control, and PFS. PRRT is well suited for treatment of NENs given that target SSTR is uniquely highly upregulated in tumor tissue. There are few off-target effects, and the overall PRRT is well tolerated compared to other cancer-directed treatments. Further research is needed to better understand how to optimize PRRT dosing and schedules to improve safety in the setting of potential hematologic toxicity. In addition, future studies using novel radionuclide agents including alpha-emitter radioligand therapy and combination therapy with PRRT with other radiosensitizing agents hold strong potential to further outcomes for patients with NENs. Last but not least, theranostics in neuroendocrine oncology is now moving beyond SSTR targeting of well-differentiated neuroendocrine tumors and exploring novel targets such as DLL-3 in high-grade neuroendocrine neoplasms (NCT06736418). The development of theranostics for patients with NENs has paved the way for an entire new industry of radioligand therapy, which is now expanding beyond the treatment of NETs to other solid tumor types leveraging targets such as FAP, HER2, GRPR, and GPC3. The success of radioligand therapy has outpaced the biological understanding of these treatment modalities. When PRRT was developed, preclinical animal models focused on histopathologic changes and toxicity (129, 137). It is no longer enough to say that the effect of radioligand therapy is related to DNA damage within the tumor. We need to understand how and why DNA breaks induced by radioligands mediate improved patient outcomes for NEN patients and the impact of this therapy on the tumor immune microenvironment. Many advanced techniques exist for exploring the biology of factors that drive cancer progression across solid tumors. In order to understand and push the field further, these techniques should be applied to tissue analysis of NENs from patients undergoing radioligand treatment to understand drivers of response and treatment resistance. The best way to identify rational targets for further drug development, both with specific alpha-emitters and with future combination therapies, is to truly understand the disease biology of WD NENs and their response to radioligand therapy.

## Declaration of interest

AC is a consultant to TerSera, Crinetics, Curioum, ITM, and Novartis; research support and consultant to Seneca Therapeutics; and research support to BMS and Clovis. The other authors declare that they do not have any financial or potential conflicts of interest. 

## Funding

This research did not receive any specific grant from any funding agency in the public, commercial, or not-for-profit sector.
